# Retrospective cohort study on factors associated with mortality in high-risk pediatric critical care patients in the Netherlands

**DOI:** 10.1186/s12887-019-1646-9

**Published:** 2019-08-06

**Authors:** Carin W. Verlaat, Nina Wubben, Idse H. Visser, Jan A. Hazelzet, SKIC (Dutch collaborative PICU research network), Dick van Waardenburg, Nicolette A. van Dam, Nicolaas J. Jansen, Marc van Heerde, Matthijs de Hoog, Martin Kneyber, Maaike Riedijk, Johannes van der Hoeven, Joris Lemson, Mark van den Boogaard

**Affiliations:** 1 Radboud Institute for Health Sciences, Department of Intensive Care Medicine Radboud, university medical center, Internal post 709, P.O. box 9101, 6500HB Nijmegen, The Netherlands; 2 Department of intensive care, Radboud university medical center, Nijmegen, the Netherlands; 3 researcher Dutch Pediatric Intensive Care Evaluation, Department of Pediatric Intensive Care, Erasmus University Medical Center - Sophia Children’s Hospital, Rotterdam, the Netherlands; 4 department of Public Health, Erasmus University Medical Center, Rotterdam, the Netherlands

**Keywords:** Child, Critical care, Mortality, Outcome assessment (healthcare)

## Abstract

**Background:**

High-risk patients in the pediatric intensive care unit (PICU) contribute substantially to PICU-mortality. Complex chronic conditions (CCCs) are associated with death. However, it is unknown whether CCCs also increase mortality in the high-risk PICU-patient. The objective of this study is to determine if CCCs or other factors are associated with mortality in this group.

**Methods:**

Retrospective cohort study from a national PICU-database (2006–2012, *n* = 30,778). High-risk PICU-patients, defined as patients < 18 years with a predicted mortality risk > 30% according to either the recalibrated Pediatric Risk of Mortality-II (PRISM) or the Paediatric Index of Mortality 2 (PIM2), were included. Patients with a cardiac arrest before PICU-admission were excluded.

**Results:**

In total, 492 high-risk PICU patients with mean predicted risk of 24.8% (SD 22.8%) according to recalibrated PIM2 and 40.0% (SD 23.8%) according to recalibrated PRISM were included of which 39.6% died. No association was found between CCCs and non-survival (odds ratio 0.99; 95% CI 0.62–1.59). Higher Glasgow coma scale at PICU admission was associated with lower mortality (odds ratio 0.91; 95% CI 0.87–0.96).

**Conclusions:**

Complex chronic conditions are not associated with mortality in high-risk PICU patients.

**Electronic supplementary material:**

The online version of this article (10.1186/s12887-019-1646-9) contains supplementary material, which is available to authorized users.

## Background

Patients with a high predicted mortality risk in the pediatric intensive care unit (PICU) are a challenge to the clinical team. The relatively small subset of these patients contributes substantially to the number of non-survivors and to PICU-resources. Around 1% of the PICU-admissions in the Australian and New Zealand Paediatric Intensive Care Registries (ANZPIC) has a predicted mortality risk between 30 and 100%, but this small cohort contributes to one third of all deaths [1–3].

Complex chronic conditions (CCCs) are associated with prolonged length of stay in PICU patients, unplanned readmissions and death [4, 5]. A CCC is defined as ‘*any medical condition that can be reasonably expected to last at least 12 months (unless death intervenes) and to involve either several different organ systems or 1 organ system severely enough to require specialty pediatric care and probably some period of hospitalization in a tertiary care center*’ [6]. There are many CCCs in several organ systems. Examples are spinal cord malformations, cystic fibrosis, hypoplastic left heart syndrome, extreme immaturity, metabolic disorders, etc. [7] Besides CCCs there are so called ‘noncomplex chronic conditions’ (NCCCs), diagnoses that could be expected to last > 12 months but not meeting the additional CCC criteria. Examples of NCCCs are asthma, atrial septal defect, obesity, etc. [4]. The prevalence of CCCs among hospitalized patients and among PICU patients is increasing [4]. Only few CCCs are incorporated in severity-of illness models like Paediatric Index of Mortality (PIM (2,3)) and Pediatric Risk of Mortality (PRISM (II, III, IV) [4, 8–12]. In low-risk PICU-patients (patients with predicted mortality risk < 1%) CCCs and unplanned admissions are associated with death (OR 3.29, 95% CI 1.97–5.50) [13, 14]. It is unknown whether CCCs increase mortality in the high-risk PICU patient as well.

Therefore, the aim of the present study is to determine if CCCs or other identifiable factors are associated with death in high-risk PICU-patients.

## Methods

### Study population

Patients were derived from a national PICU database containing data from all pediatric intensive care departments in the Netherlands (2006–2012, *n* = 30,778); the ‘PICE-registry’ [13, 15]. The same cohort was used in a previous study on low-risk PICU-patients [13]. Patients < 18 years old with a high predicted mortality risk were included in the study. High-risk was defined as a predicted mortality risk > 30% according to either the PRISM II (referred to as PRISM) or the PIM2 risk score [9, 10]. In this study, as described before, both models were recalibrated to predict the overall mortality in the total population in this particular 6-year period without altering the relative weights of risk factors in the models and thus retaining the discriminative power of the original models [13, 15].

Patients who were already dead before PICU admission (e.g., patients admitted for organ transplantation already being brain-dead) or patients admitted for palliative care, patients dying within 2 h of PICU admission, and patients transferred to another ICU during their PICU treatment were excluded from the study. Data of patients that did not pass quality control during local site audit visits and were excluded from the annual reports were also excluded from the study [13]. Patients with a cardiac arrest prior to PICU admission were excluded due to possible bias of the results [16, 17].

### Design

Retrospective cohort study based on data prospectively collected in a national registry.

#### Risk variables and data-handling

Variables that were analysed represented many aspects of the PICU stay, including admission characteristics, physiological state, diagnoses and outcome. Non-survivors were defined as patients who died in the PICU. The ANZPIC diagnostic code list was used in the PICE-registry [18]. Patients were classified as patients with a CCC if either the primary diagnosis, underlying diagnosis or first additional diagnosis was a CCC [6, 7]. Patients were classified as having a NCCC if the primary diagnosis, underlying diagnosis or first additional diagnosis was a diagnosis defined as a NCCC. A modified Feudtner’s list was used to classify diagnoses into CCC or NCCC [4, 6, 7, 18]. ANZPIC diagnoses not appearing on these lists were classified according to expert opinion (C.V. and J.L.). The list of CCC-diagnoses was recently published [13]. Definitions of ‘Admission outside office hours’, ‘readmission’ and ‘specialized transport’ were published previously [13]. The data were checked for non-valid data. Illogical and impossible values that surpassed physiologic threshold values were excluded if the value likely resulted from a typo or measurement error, as described before. (Examples of typo/measurement errors: diastolic blood pressure > 400 mmHg, low paO2 in combination with cyanotic congenital heart disease which by definition should be excluded from PRISM score.) [13].

### Statistical analysis

Depending on distribution, continuous variables were tested using an independent T test or Mann-Whitney U test. For dichotomous variables, chi-square test or, in case of small expected frequencies, Fisher’s exact test was used. To adjust for multiple testing, Bonferroni correction was performed and differences were considered statistically significant if *p*-value was < 0.001.

For the multivariable logistic regression analysis, only risk factors that were present at the time of admission were included in the regression analysis. Because the selection of the study population was based on PIM2 and PRISM scores, predictors from these scores were not included in the multivariable logistic regression analysis, except for the Glasgow Coma Scale (GCS) at admission.

Statistical analyses were carried out using IBM SPSS Statistics Version 22.1.

## Results

### Population characteristics

**Fig. 1:**
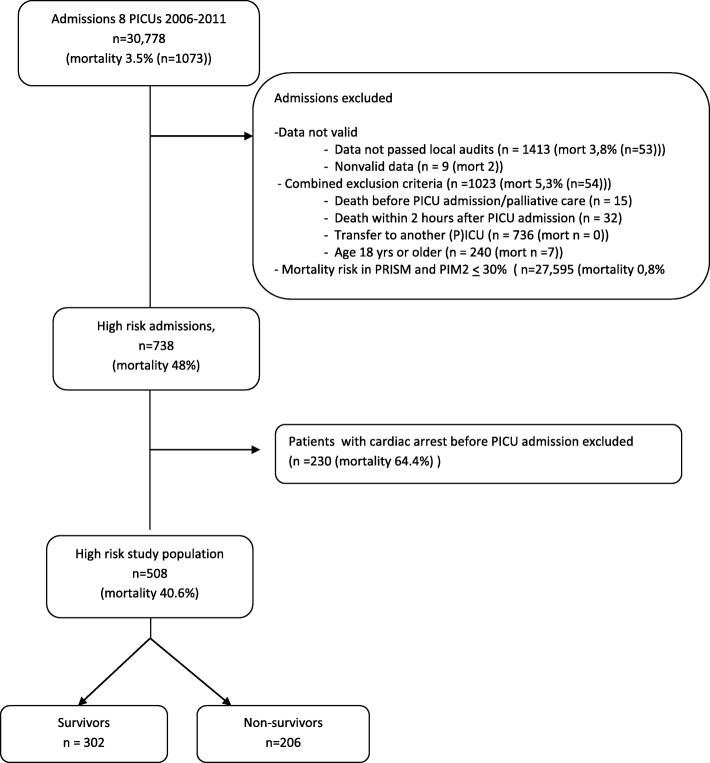
Flowchart of the population

In total, there were 30,778 admissions of which 738 patients were high-risk patients (Fig. 1, Additional file 1: Table S3). After excluding patients with cardiac arrest before PICU admission, a total of 492 high-risk patients was included with a mortality rate of 39.6%. The mean predicted mortality risk of these 492 patients was 24.8% (SD: 22.8%) according to the recalibrated PIM2 and 40.0% (SD: 23.8%) according to the recalibrated PRISM. The majority of the high-risk patients had an unplanned admission for medical reasons.

### Analysis of differences

Baseline characteristics are shown in Table 1. The median GCS at time of admission was significantly higher in survivors compared to non-survivors (median 15 vs. median 12, respectively; *p* < 0.001). Both PRISM and PIM2 mortality risks were significantly lower in survivors compared to non-survivors. Ventilator-days and length of stay were longer in survivors compared to non-survivors. No other significant differences were found.

**Table 1:** Population characteristics and differences between high-risk survivors and non-survivors

Characteristic	Survivors *n* = 297	Non-survivors *n* = 195	*p* value
Male	179 (60.3)	105 (53.8)	0.16
Age < 12 months	161 (54.2)	90 (46.2)	0.08
Unplanned admission	275 (92.6)	182 (93.3)	0.76
Medical admission	227 (76.4)	146 (74.9)	0.69
Readmission < 48 h	4 (1.3)	3 (1.5)	0.86
Admission outside office hours	151 (50.8)	96 (49.2)	0.73
Mode of transport upon admission			
None	159 (53.5)	104 (53.3)	0.97
Non-specialized transport	52 (17.5)	20 (10.3)	0.03
Specialized transport	107 (36.0)	84 (43.1)	0.12
Season of admission			
Winter	74 (24.9)	46 (23.6)	0.74
Spring	64 (21.5)	51 (26.1)	0.24
Summer	59 (19.8)	50 (25.6)	0.13
Autumn	100 (33.7)	48 (24.6)	0.03
Recovery as reason for PICU admission	22 (7.4)	15 (7.7)	0.91
PRISM recalibrated mortality risk, median [IQR]	0.36 [0.15–0.48]	0.44 [0.31–0.66]	< 0.001
PIM2 recalibrated mortality risk, median [IQR]	0.14 [0.05–0.34]	0.21 [0.09–0.46]	< 0.001
Patients with			
PRISM > 30% (and PIM < 30%)	190 (64.0)	115 (59.0)	0.26
PIM2 > 30% (and PRISM < 30%)	89 (30.0)	43 (22.1)	0.05
PRISM and PIM2 > 30%	18 (6.1)	37 (19.1)	< 0.001
Chronic conditions			
No chronic condition	82 (27.6)	68 (34.9)	0.09
NCCC	19 (6.4)	7 (3.6)	0.17
CCC	196 (66.0)	120 (61.5)	0.31
Diagnose groups			
Trauma	9 (3.0)	16 (8.2)	0.01
Cardiovascular	30 (10.1)	22 (11.3)	0.68
Neurological	30 (10.1)	31 (15.9)	0.06
Respiratory	79 (26.6)	29 (14.9)	0.002
Renal	2 (0.7)	1 (0.5)	0.82
Gastrointestinal	14 (4.7)	12 (6.2)	0.49
Post procedure diagnosis	45 (15.2)	32 (16.4)	0.71
Miscellaneous	88 (29.6)	52 (26.7)	0.48
Glasgow Coma Scale at admission	15 [9–15]	12 [3–15]	< 0.001
Mechanically ventilated (*n* = 660)	260 (91.5)	178 (97.8)	0.01
Outcome			
Number of days mechanically ventilated, median [IQR]	7 [4–13]	3 [2–7]	< 0.001
Length of stay PICU, median [IQR]	12 [7–21]	3 [2–7]	< 0.001

Data are presented as n (%), unless mentioned otherwise

[IQR] is defined as interquartile range: [25th percentile – 75th percentile]

*NCCC* non-complex chronic condition, *CCC* complex chronic condition

The physiological parameters are the most abnormal values collected in the first 24 h after admission

### Factors associated with survival

Higher GCS at admission was associated with lower mortality (OR 0.91; 95% CI 0.87–0.96) (Table 2). No association was found between CCCs and non-survival (OR 0.99; 95% CI 0.62–1.59). No other factors were associated with mortality. Results from the unadjusted ORs are shown in (Additional file 1: Table S3).

**Table 2:** Variables associated with non-survival in the high-risk group

Factor	OR	95% CI
Male	0.75	0.51–1.12
Age < 1 yr.	0.84	0.56–1.27
Specialized transport	1.24	0.82–1.88
Admission outside office hours	0.79	0.53–1.17
Season		
Winter	Ref	
Spring	1.29	0.74–2.25
Summer	1.53	0.87–2.70
Autumn	0.84	0.49–1.43
Chronic conditions		
No chronic condition	Ref	
CCC	0.99	0.62–1.59
NCCC	0.53	0.19–1.45
Diagnose subgroups		
Trauma	Ref	
Cardiovascular	0.91	0.30–2.77
Neurological	1.06	0.48–2.33
Respiratory	0.85	0.39–1.87
Renal	0.70	0..36–1.36
Gastrointestinal	0.61	0.05–7.79
Post procedure	0.77	0.42–1.44
Miscellaneous	1.38	0.54–3.52
Glasgow coma scale at admission	0.91	0.87–0.96

*NCCC* non-complex chronic condition, *CCC* complex chronic condition

Results from the unadjusted ORs are shown in Additional file 1: Table S3

## Discussion

In this large retrospective cohort study in high-risk PICU patients, complex chronic conditions were not associated with mortality.

This is different compared to our previous study looking into low-risk admissions, where CCCs were associated with increased mortality [13]. In a general PICU-population, without risk stratification, a similar association was found [4]. Although some CCCs (for example: leukemia, hypoplastic left heart syndrome) are incorporated in the PIM2, the majority of CCCs is not part of the risk models. Having a chronic disease is often not reflected in physiological values and therefore not shown as a higher mortality risk. CCCs can be very heterogeneous. Some CCCs might be associated with death in the PICU (e.g. a patient with a complex heart disorder) while other CCCs are not lethal but may have impact on other outcome parameters like functional outcome. Furthermore, it’s possible that some patients with CCCs may be refused PICU admission and thus not contribute to the overall PICU mortality. We did not investigate this and therefore this statement is conjecture. In true high-risk patients other factors like the GCS have a clearer influence on mortality for patients with CCCs.

Our study has several limitations. First, an arbitrary choice was made for the definition of high-risk patients, using a combination of PIM2 and PRISM scores with a certain cut-off point. Both models use different predictors and different time windows to calculate their scores and do not give the same result. Because in the Dutch PICE registry both models are used and no model is superior to another, we used a combination of both models. Using only one model instead of a combination might underestimate a cohort of high-risk patients. Only a minority had a mortality risk of > 30% in both models. Mean predicted mortality was higher according to PRISM compared to PIM2. However, if only PRISM model had been used to detect high-risk patients, roughly a third of the high-risk cohort would not have been detected.

Third, an older version of the PRISM was used, dating from 1988 [10]. If the original PRISM model would have been used without recalibration, the predicted mortality would have been overestimated. However, because the PRISM was recalibrated to fit, it is a good predictor of mortality [15].

Fourth, no factors which are part of the PIM2/PRISM models were used for the multivariable logistic regression analysis, with the exception of the GCS at admission. The GCS at admission is not incorporated in the PIM2 model but is indirectly part of the PRISM score as a dichotomous variable. If the GCS within the first 24 h is less than 8, the PRISM score increases. However, a mild decrease in GCS such as GCS between 8 and 10 does not increase PRISM score, although there might be a serious neurological condition. We found a significant and clinically important lower GCS in non-survivors. This difference could not be explained by cardiac arrest patients. Therefore we decided to add the GCS as a continuous variable in the analysis.

## Conclusions

Complex chronic conditions are not associated with mortality in PICU patients with a high predicted mortality-risk, in contrast to low-risk PICU patients. We recommend to explore the role of CCCs in (PICU) patients with different risk profiles further. Higher Glasgow coma scale at PICU admission was associated with lower mortality.

## Additional file

Additional file 1: **Table S3.** Variables associated with mortality survival in the high-risk group. (DOCX 12 kb)

## Data Availability

The data that support the findings of this study were used under license from the national PICU database containing data from all pediatric intensive care departments in the Netherlands (‘PICE registry’) for the current study, and so are not publicly available. Limited data are available from the author on reasonable request.

## Abbreviations

ANZPIC: Australian and New Zealand Paediatric Intensive Care Registries
CCCs: Complex chronic conditions
CGS: Glasgow coma scale
NCCC: Non-complex chronic condition
PICE registry: Dutch pediatric intensive care registry (‘Pediatrische Intensive Care Evaluatie’)
PICU: Pediatric intensive care unit
PIM: Paediatric Index of Mortality
PRISM: Pediatric Risk of Mortality
