# Supplementary material for: Retrospective cohort study on factors associated with mortality in high-risk pediatric critical care patients in the Netherlands
Source: BMC Pediatr. 2019 Aug 6;19:274. doi: 10.1186/s12887-019-1646-9 (PMC6683538; doi:10.1186/s12887-019-1646-9)
Supplement: Supplementary file 1 — Table S3. Variables associated with mortality survival in the high-risk group. (DOCX 12 kb) [file 12887_2019_1646_MOESM1_ESM.docx]

**Table 3 : Variables associated with mortality survival in the high-risk group**

| **Factor** | **Crude OR** | **95% CI** | **Adjusted OR** | **95% CI** |
| --- | --- | --- | --- | --- |
| Male | 0.77 | 0.53-1.11 | 0.75 | 0.51-1.12 |
| Age < 1 yr | 0.72 | 0.50-1.04 | 0.84 | 0.56-1.27 |
| Specialized transport | 1.34 | 0.93-1.94 | 1.24 | 0.82-1.88 |
| Admission outside office hours | 0.94 | 0.65-1.35 | 0.79 | 0.53-1.17 |
| Season  -Winter  -Spring  -Summer  -Autumn | Ref  1.28  1.36  0.77 | 0.76-2.16  0.81-2.31  0.47-1.28 | Ref  1.29  1.53  0.84 | 0.74-2.25  0.87-2.70  0.49-1.43 |
| Chronic conditions  - No chronic condition  - CCC  - NCCC | Ref  0.74  0.44 | 0.50-1.09  0.18-1.12 | Ref  0.99  0.53 | 0.62-1.59  0.19-1.45 |
| Diagnose subgroups  - Trauma - Cardiovascular - Neurological - Respiratory - Renal - Gastrointestinal  - Post procedure  - Miscellaneous | Ref  0.41  0.58  0.21  0.28  0.48  0.40  0.33 | 0.15-1.10  0.22-1.52  0.08-0.52  0.02-3.55  0.16-1.48  0.16-1.02  0.14-0.81 | Ref  0.91  1.06  0.85  0.70  0.61  0.77  1.38 | 0.30-2.77  0.48-2.33  0.39-1.87  0.36-1.36  0.05-7.79  0.42-1.40  0.54-3.52 |
| Glasgow coma scale at admission | 0.91 | 0.87-0.94 | 0.91 | 0.87-0.96 |

OR = odds ratio, NCCC = non-complex chronic condition, CCC = complex chronic condition
